# PLA2G7/PAF-AH as Potential Negative Regulator of the Wnt Signaling Pathway Mediates Protective Effects in BRCA1 Mutant Breast Cancer

**DOI:** 10.3390/ijms24010882

**Published:** 2023-01-03

**Authors:** Yue Liao, Susann Badmann, Fabian Kraus, Nicole Elisabeth Topalov, Doris Mayr, Thomas Kolben, Anna Hester, Susanne Beyer, Sven Mahner, Udo Jeschke, Fabian Trillsch, Bastian Czogalla, Alexander Burges

**Affiliations:** 1 Department of Obstetrics and Gynecology, University Hospital, LMU Munich, 81377 Munich, Germany; liaoyue1910@gmail.com (Y.L.); susann.badmann@hotmail.de (S.B.); fabian.kraus@med.uni-muenchen.de (F.K.); nicole.topalov@med.uni-muenchen.de (N.E.T.); thomas.kolben@med.uni-muenchen.de (T.K.); anna.hester@med.uni-muenchen.de (A.H.); susanne.beyer@med.uni-muenchen.de (S.B.); sven.mahner@med.uni-muenchen.de (S.M.); udo.jeschke@med.uni-muenchen.de (U.J.); fabian.trillsch@med.uni-muenchen.de (F.T.); alexander.burges@med.uni-muenchen.de (A.B.); 2 Xiangyang No.1 People´s Hospital, Hubei University of Medicine, Xiangyang 441000, China; 3 Institute of Pathology, Faculty of Medicine, LMU Munich, 80337 Munich, Germany; doris.mayr@med.uni-muenchen.de; 4 Department of Obstetrics and Gynecology, University Hospital Augsburg, 86156 Augsburg, Germany

**Keywords:** platelet-activating factor acetylhydrolase (PAF-AH; *PLA2G7*), BRCA1 mutant breast cancer, β-catenin, Wnt signaling, prognosis

## Abstract

Past studies have confirmed that aberrant activation of the Wnt/β-catenin signaling is associated with tumorigenesis and metastasis in breast cancer, while the role of platelet-activating factor acetylhydrolase (PLA2G7/PAF-AH) in this signaling pathway remains unclear. In this study, we analyze the functional impact of PAF-AH on BRCA1 mutant breast cancer and explore its relationship to the Wnt signaling pathway. By performing immunohistochemistry, PAF-AH expression and β-catenin expression were examined in both BRCA1 WT and BRCA1 mutant breast cancer specimens. The BRCA1 mutant breast cancer cell line HCC1937 was used for in vitro experiments to assess the impact of PAF-AH on cellular functions. The intracellular distribution of β-catenin depending on PLA2G7/PAF-AH expression was investigated by immunocytochemistry. Significantly higher nuclear expression levels of PAF-AH were found in BRCA1 mutant tissue specimens than in BRCA1 WT samples. Cell viability, proliferation, and the motility rate of HCC1937 were significantly enhanced after PLA2G7 silencing, which indicated a protective role of PAF-AH in breast cancer. Nuclear PAF-AH expressed correlatedly with membranous β-catenin. PLA2G7 silencing provoked the β-catenin translocation from the membrane to the nucleus and activated Wnt signaling downstream genes. Our data showed a protective effect of high PAF-AH expression in BRCA1 mutant breast cancer. PAF-AH may achieve its protective effect by negatively regulating the Wnt pathway. In conclusion, our research sheds new light on the regulatory pathways in BRCA1 mutant breast cancer.

## 1. Introduction

With an estimated annual incidence of 1.38 million new cases worldwide, breast cancer (BC) is the most prevalent malignancy in women. It is also the most common cause of death in female cancer patients globally [1]. In contrast to the favorable prognosis of many early BC patients, the survival rate for advanced BC is still low. In part, this can be attributed to limitations of screening variables [2]. Thus, in addition to the classic prognostic factors (such as patient age, tumor stage, grade, histological subtype, etc.) [3,4,5,6], the search for new biomarkers must be advanced. These molecules are not only supposed to serve as prognostic predictors, but also identify high-risk patients. Given the histopathological differences between the breast cancer 1 gene (BRCA1) mutated and non-BRCA1-related tumors, a widely recognized prognostic biomarker for BRCA1-positive BC remains to be found.

The BRCA1 gene is located on human chromosome 17q21, on the breast cancer 2 gene (BRCA2) on 13q12. As major gatekeepers for genome integrity, both BRCA1 and BRCA2 encode proteins involved in DNA double-strand break (DSB) repair mechanisms [7,8,9] and are, therefore, susceptibility genes for breast and ovarian cancer (OC) [10,11]. DSB repair mechanisms have two mainly categories: homologous recombination (HR) and non-homologous end-joining (NHEJ) [10,12,13,14]. BRCA1 is involved in both HR and NHEJ repair systems [15,16]. In addition, BRCA1 gene products are also involved in DSB by directly interacting with enzymes that modify chromatin and DNA structure [17,18]. Consistently, carriers of BRCA1 loss of function mutations have a lifetime risk of 70–80% for breast and 44% for ovarian cancer [19]. Thus, BRCA1 gene status screening is vital for breast and ovarian cancer prevention strategies.

PAF-AH is an enzyme with a variety of biological effects. To date, three types of PAF-AH have been found in mammalian tissues: intracellular types I and II and plasma type. Type I PAF-AH is a G-protein-like complex consisting of two catalytic subunits (alpha1 and alpha2) and a regulatory beta subunit. Type II PAF-AH is a single polypeptide and shows significant sequence homology with plasma PAF-AH, and both may act as scavenger proteins for oxidized phospholipids. This study focuses on the biological features of plasma PAF-AH, which degrades the platelet-activating factor (PAF) [20,21,22,23]. PAF is a phospholipid mediator secreted into the tumor microenvironment by circulating cells and cancer cells that mediates its effect through a specific G-protein-coupled receptor (PAFR). Evidence suggests that the PAF–PAFR interaction is involved in oncogenic transformation, anti-apoptosis, metastasis, and angiogenesis [24,25,26,27]. Therefore, PAF signaling cascade becomes a potential target for future BC therapies. However, PAF-AH’s molecular function seems to be dependent on the cancer type. On the one hand, high-level PAF-AH expression is associated with aggressive disease and poor prognosis in prostate cancer and triple-negative BC [28,29]. On the other hand, there is evidence of reduced tumor growth and prolonged survival in mouse models of Kaposi’s sarcoma and melanoma with PAF-AH overexpression [30]. Through our recent results, our team identified PAF-AH as an independent positive prognostic factor for overall survival in OC patients [31].

Including breast and ovarian cancer, another well-established driver of tumorigenesis and cancer progression in many human cancers is the aberrantly activated Wnt signaling pathway [32,33,34]. As a critical downstream effector of the canonical Wnt signaling pathway, β-catenin translocates and accumulates in the nucleus, activating Wnt response genes [35,36,37]. Recent studies reported a complete loss of membrane-bound β-catenin in invasive lobular breast carcinomas [38]. Similarly, in invasive ductal carcinoma, the loss of membranous β-catenin evokes the activation of the canonical Wnt pathway and correlates with poor outcomes in BC patients [39,40]. Moreover, PAF is highly expressed in BC cells and positively regulates the Wnt signaling pathway as a cofactor of the β-catenin transcription complex [41,42]. Our previous results indicate a negative regulatory impact of PAF-AH on the Wnt/β-catenin signaling pathway in BRCA1 mutated OC [31]. *PLA2G7* silencing in BRCA1 mutant OC cells reduced the expression of membranous β-catenin, but led to a strong upregulation of its nuclear expression. The resulting increase in Wnt signal transduction might be one of the key drivers of OC progression, qualifying PAF-AH as a potential therapeutic target to slow down OC progression. In this study, we aim to characterize PAF-AH expression in BRCA1 mutated BC as well as its regulatory effects on the Wnt signaling pathway.

## 2. Results

### 2.1. Nuclear PAF-AH Is Highly Expressed in BRCA1 Mutant BC Tissue and Correlates Positively with Membranous β-Catenin

A total of 121 BC cases were studied for PAF-AH expression by immunohistochemistry (IHC), and 44 cases were available for β-catenin detection. Out of 121 cases, 39 (32.2%) were positive for nuclear PAF-AH, 103 (85.1%) for cytoplasmic PAF-AH, and 21 (17.3%) for both. Median (range) immunoreactivity scores (IRS) for PAF-AH in the nucleus and cytoplasm were 0 (0, 9) and 3 (0, 12). Membranous expression of β-catenin was positive in 39 (88.6%) out of 44 cases with a median (range) IRS of 6 (0, 12), while 5 (11.4%) cases showed a nuclear staining of β-catenin with a median IRS of 3 (0, 6).

**Figure 1:**
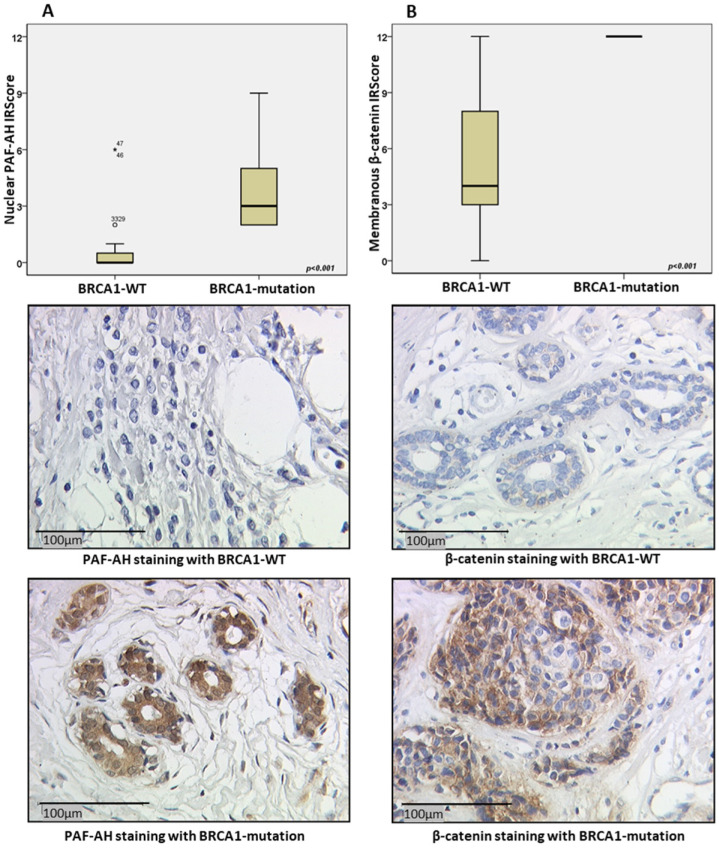
BRCA1 mutation carriers show stronger nuclear PAF-AH and membranous β-catenin staining intensity. Compared to BRCA1 WT, cases with BRCA1 mutation (n = 22; median IRScore = 3) expressed significantly more nuclear PAF-AH, the outliers in BRCA1-WT were indicated with small circle and asterisk (*p* < 0.001) (**A**). Among all cases with membranous β-catenin expression, BRCA1 mutation carriers (n = 14; median IRScore = 12) showed higher membranous expression than those without BRCA1 mutation (n = 19; median IRScore = 4) (*p* < 0.001) (**B**).

An evaluation regarding the BCRA1 mutation status revealed that patients with BRCA1 mutation expressed higher nuclear PAF-AH levels than patients with BRCA1 WT (*p* < 0.001, Figure 1A). The same trend was noticed for membranous β-catenin expression (*p* < 0.001, Figure 1B). There was no significant difference of cytoplasmic PAF-AH regarding the BRCA1 mutation status.

A strong positive correlation between nuclear PAF-AH and membranous β-catenin was found (Table 1).

### 2.2. Only BRCA1 Negative Cell Line HCC1937 Shows Relevant Expression of PLA2G7/PAF-AH

**Figure 2:**
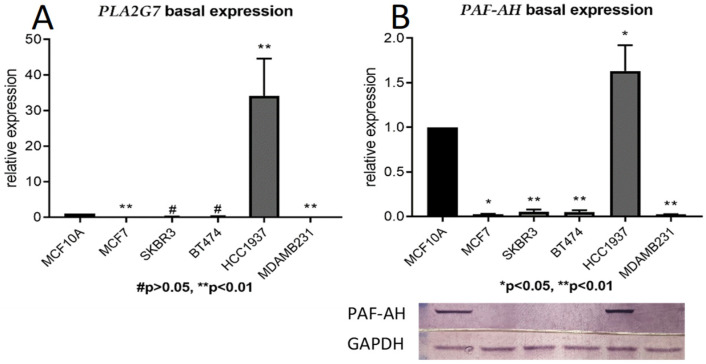
Only the BRCA1 negative BC cell line HCC1937 shows a relevant expression of PLA2G7/PAF-AH. Basal mRNA (qPCR; (**A**)) and protein (Western blot analysis; (**B**)) expression of PLA2G7/PAF-AH in five BC cell lines were compared to the expression in the benign breast cell line MCF10A. Significant results are indicated by asterisks (*: *p* ≤ 0.05, **: *p* ≤ 0.01), and non-significant results by diamonds (#: *p* > 0.05).

The basal mRNA and protein expression of PLA7G7/PAF-AH of five BC cell lines were compared to the breast epithelial cell line MCF10A. Both PLA2G7 expression on the mRNA level (*p* < 0.01; Figure 2A) and PAF-AH expression on the protein level (*p* < 0.001; Figure 2B) were significantly increased in the BRCA1 mutant BC cell line HCC1937 compared to MCF10A and other BC cell lines. On the protein level, it is striking that in other breast cancer cell lines, PAF-AH expression is even significantly reduced compared to benign cells.

### 2.3. PLA2G7 Downregulation Enhanced Viability, Proliferation, and Motility of HCC1937 Cells

**Figure 3:**
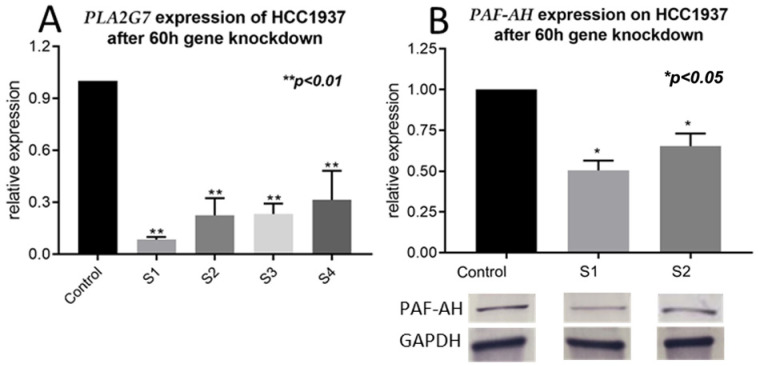
Successful downregulation of PLA2G7/PAF-AH by siRNA knockdown. The efficiency of siRNA knockdown was investigated by qPCR (**A**) and western blot analysis (**B**). The best knockdown of PLA2G7 was achieved after an incubation time of 60 h (*p* < 0.01; **A**). Concordant to the result on RNA level, western blot analysis showed a decrease in protein expression (*p* < 0.05), confirming a successful downregulation (**B**).

To assess the functional role of PLA2G7/PAF-AH in BC pathogenesis and progression, in vitro experiments were performed. Since the BRCA1 negative cell line HCC1937 showed the highest levels of PAF-AH, this cell line was used for functional assays after gene knockdown. Firstly, siRNA was transfected into HCC1937 for PLA2G7 silencing. A successful downregulation of PLA2G7 and its protein PAF-AH was confirmed by qPCR and western blot analysis (Figure 3).

**Figure 4:**
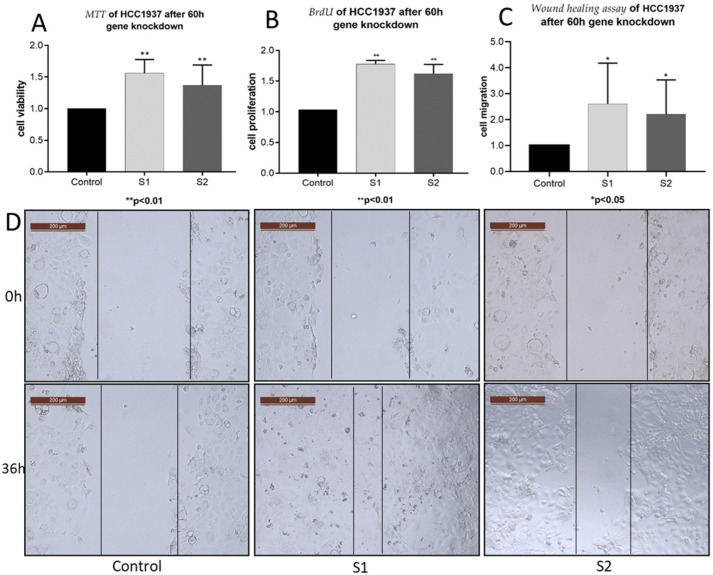
PLA2G7/PAF-AH downregulation causes cancer progression by an activation of cell viability, proliferation, and migration. In MTT assays, the viability of PLA2G7 silenced HCC1937 significantly increased (*p* < 0.01) (**A**). BrdU results (**B**) showed an increased proliferation capacity (*p* < 0.01), and wound healing assays indicated an augmented migration ability of PLA2G7 downregulated HCC1937 cells (*p* < 0.05) (**C**,**D**). All assays were conducted 60 h after transfection with siRNA-PLA2G7 and scaled to a control treated with scrambled siRNA. Significant results are indicated by asterisks (*: *p* ≤ 0.05) and double asterisks (**: *p* ≤ 0.001).

After *PLA2G7* silencing, the viability of HCC1937 increased (Figure 4A). Furthermore, higher absorbance values of transfected HCC1937 cells in BrdU assay in comparison with the control group (Figure 4B) indicated an increased proliferation rate of BC cells by *PLA2G7* gene silencing. Wound healing assays elicited significantly improved migration ability by *PLA2G7* knockdown (Figure 4C,D). These results summarily demonstrate that *PLA2G7* silencing favors cancer progression by an increase in viability, proliferation, and migration of BC cells.

### 2.4. The Intracellular Distribution of β-Catenin Changed from High Expression in the Membrane to High Expression in the Nucleus by PLA2G7 Knockdown

**Figure 5:**
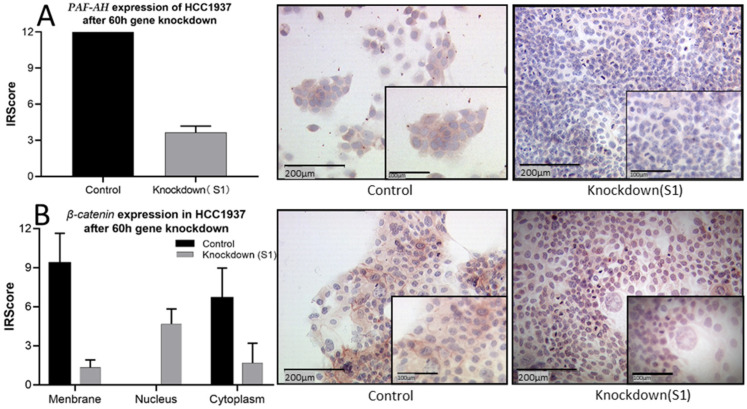
Activation of the Wnt signaling pathway by PLA2G7 silencing. After 60 h transfection with siRNA-PLA2G7, the expression of PAF-AH was significantly suppressed (**A**), while the expression of β-catenin shifted from the membrane to the nucleus, activating Wnt downstream genes (**B**). In addition, HCC1937 cells show a higher mitotic activity after gene knockdown.

After demonstrating the functional changes depending on *PLA2G7* expression, we evaluated how PAF-AH affects the Wnt signaling pathway. Based on the correlation of PAF-AH and β-catenin found in IHC, a series of immunocytochemistry was carried out to investigate PAF-AH and β-catenin expression in HCC1937 cells. As expected, PAF-AH staining was downregulated by *PLA2G7* silencing (Figure 5A). Interestingly, the distribution pattern of β-catenin was also changed by *PLA2G7* gene knockdown. While membranous expression of β-catenin was weakened compared to the control group, the nuclear expression was enhanced (Figure 5B), and will probably activate the Wnt downstream genes. In addition, HCC1937 cells showed a higher mitotic activity after *PLA2G7* gene knockdown, consistent with the results of the functional assays.

## 3. Discussion

In this study, we investigated the role of *PLA2G7*/PAF-AH and its potential impact on the Wnt signaling pathway in BC. Significantly higher nuclear PAF-AH expression was detected in BRCA1 mutated BC specimens compared to BRCA-WT (Figure 1). Consistently, relevant gene and protein expression of *PLA2G7*/PAF-AH were only shown in BRCA1 mutant cell line HCC1937 (Figure 2A,B), while other BC cell lines show almost no PAF-AH expression compared to benign breast cells. Functional analyses of cell viability, proliferation, and migration demonstrated a protective effect of PAF-AH in BC (Figure 3). Furthermore, we demonstrated a strong positive correlation of nuclear PAF-AH and membranous β-catenin expression (Table 1). A changed distribution pattern of β-catenin within the cellular departments in BRCA1 mutant BC cells caused by *PLA2G7* gene knockdown confirms a regulatory influence of *PLA2G7*/PAF-AH on the Wnt signaling pathway. Membrane expression of β-catenin was decreased, while nuclear expression was up-regulated by *PLA2G7* silencing (Figure 4), enabling the activation of Wnt downstream genes. Thus, we hypothesize a negative regulatory role of PAF-AH on the Wnt/β-catenin pathway, especially in BRCA1 mutant BC.

A previous study based on a mouse model has shown that the catalytic subunits of intracellular PAF-AH isoform IB directly modulate the Wnt signaling pathway [43]. Although PAF-AH isoforms show low sequence homology, they share the same functions in PAF catabolism and oxidative phospholipid fragmentation [44,45]. In addition to affecting the Wnt signaling pathway through PAF degradation, the functional subunits of PAF-AH might also have similar properties in regulating the Wnt signaling pathway directly.

An association between *PLA2G7*/PAF-AH and BRCA1 has already been investigated in a few studies. Hong et al. compared the differential gene expression in BRCA1-deleted mouse embryonic fibroblasts and its wild-type counterparts. They found higher *PLA2G7* expression in BRCA1-deleted mice and confirmed that BRCA1 deficiency induces protein reprogramming [46]. Furthermore, Gorrini et al. reported that BRCA1 modulates NRF2-dependent antioxidant signaling by facilitating its stability and activation [47]. *PLA2G7*, in turn, is controlled by NRF2 [48,49]. In IHC and in vitro experiments, our study also observed a strong coincidence between high PAF-AH expression and BRCA1 mutation status, especially for nuclear PAF-AH expression.

We observed that high nuclear expression of PAF-AH was closely related to membranous expression of β-catenin. An interplay between BRCA1 and the Wnt signaling pathway has been described before. While Wu et al. found an inverse correlation between Wnt signaling and BRCA1 expression in basal-like breast cancer due to epigenetic repression of BRCA1 by the Wnt effector Slug [50], Li et al. reported that the nuclear form of β-catenin was lower or absent in most BRCA1 familial breast cancer tissues compared to sporadic breast cancer or healthy tissue [51]. For BRCA1 WT, but not mutated BRCA1, a direct interaction with β-catenin on the same binding site as the ubiquitinylating enzyme was described. Consequently, the half-life of β-catenin is prolonged and the Wnt signaling pathway is active in the presence of BRCA1 WT [51].

**Figure 6:**
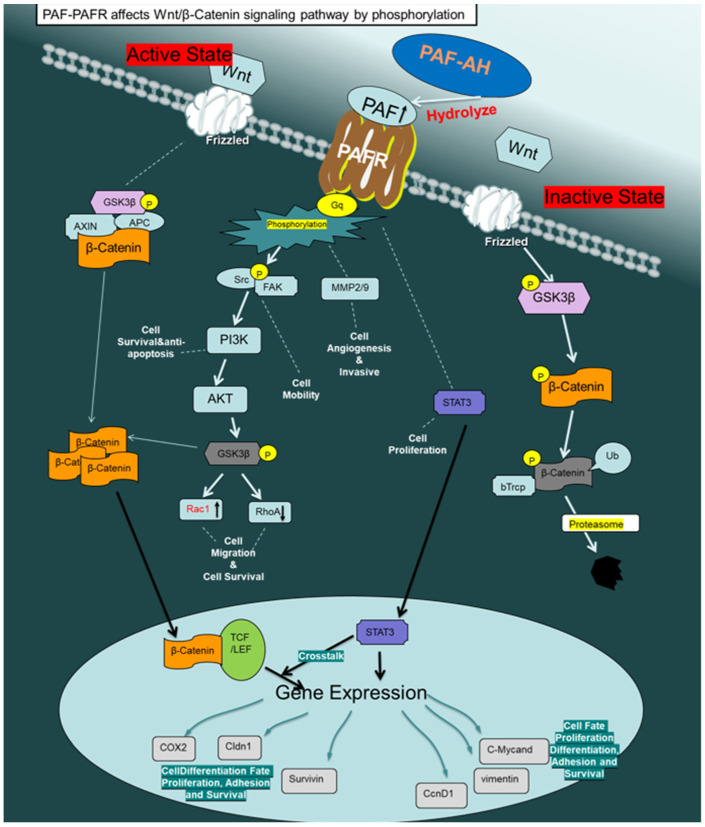
PAF-PAFR affects the Wnt/β-catenin signaling pathway by phosphorylation. The active state of Wnt/β-catenin signaling is shown on the left. The inactive state of Wnt/β-catenin signaling and degradation of β-catenin is shown on the right. PAF–PAFR signaling and cell functions changed after activation by phosphorylation (middle).

To date, it remains inconclusive whether PAF-AH not only affects the Wnt/β-catenin signaling pathway, but whether the Wnt pathway itself might also influence PAF-AH expression. Zhang et al. showed that PAFR expression in BRCA1 mutant cell lines and tissue specimens of BRCA1 mutation carriers was increased. They demonstrated that PAF/PAFR mediates malignant transition of non-malignant ovarian epithelial cells with BRCA1 mutations, inducing proliferation and anti-apoptosis through FAK/STAT phosphorylation. The study also indicated that an up-regulation of PAF-AH might be associated with the Wnt/β-catenin signaling pathway and PAF expression, once more illustrating a complex relationship between Wnt/β-catenin and PAF signaling [52] (Figure 6).

Based on our experimental results involving BC cell lines and patient specimens, we found an increased PAF-AH expression in BRCA1 mutant BC cells and identify PAF-AH as a potential negative regulator of the canonical Wnt/β-catenin pathway. By functional assays in HCC1937 cells, we show that the loss of PAF-AH leads to cancer progression with enhanced viability, proliferation, and migration. This finding underlines its protective character which we have already shown for the BRCA1 mutant OC [31]. This effect might be mediated by an inhibition of the Wnt/β-catenin pathway. However, the influence of additional signaling pathways or regulatory factors on the effects described in this study cannot be excluded and remain a matter for future research as well as the exact molecular interaction between PAF-AH and the Wnt/β-catenin signaling pathway.

## 4. Materials and Methods

### 4.1. Ethical Approval

Tumor tissue analyzed within this study had initially been collected for routine histo-pathological diagnostics. After completing the diagnostic procedures, all samples were considered for inclusion in the study. All analyses were performed according to the standards set in the declaration of Helsinki 1975. Patient data were fully anonymized and the Ethics Committee of the Ludwig-Maximilians-University (Munich, Germany) approved the study (approval number 048-08). Researchers were blinded from patient data during experimental workup.

### 4.2. Patient Specimens

This study analyzed 121 tissue samples from patients who underwent BC surgery at the Department of Obstetrics and Gynecology, Ludwig Maximilian University, between 1987 and 2009. Women with a benign breast tumor or an in situ carcinoma were excluded from the study, as were patients that had undergone neoadjuvant chemotherapy. Gynecological pathologists assessed all BC cases. Detailed information on the clinical features of patients enrolled in this study included tumor grading, histology, and staging. The clinical-pathological variables are summarized in Table 2. The mean age (± STDV) of the cohort was 50.0 ± 13.3 years (BRCA1 associated cases: 41.9 ± 10.8 years; sporadic BC: 53.7 ± 12.8 years).

### 4.3. Immunohistochemistry and Immunocytochemistry

Tissue specimens were set in formalin, embedded in paraffin, and cut into 3 µm sections in our laboratory [53]. Subsequently, the tumor slides were dewaxed in xylol, washed in 100% ethanol, incubated in methanol with 3% H_2_O_2_ for 20 min, and rehydrated in a descending ethanol gradient. The slides were cooked for 5 min in a sodium citrate buffer (pH = 6.0) consisting of 0.1 M citric acid and 0.1 M sodium citrate in distilled water. After cooling, the slides were washed in PBS and incubated for 30 min with a blocking solution to prevent non-specific binding of the primary antibodies (Reagent 1, Zytochem-Plus HRP-Polymer-Kit (mouse/rabbit)). The slides were then incubated with rabbit polyclonal anti-PAF-AH (Cayman, Polyclonal Cay-160603, 1:200 dilution, rabbit IgG, Michigan, USA), and anti-β-catenin antibodies (Diagnostic Biosystems, Polyclonal Roche-D178frzQ, 1:300 dilution, rabbit IgG, Pleasanton, CA, USA) for 16 h at 4 °C. After that, the slides were washed with PBS and incubated with the secondary antibodies or HRP-polymer complexes (Reagent 3; Zytochem-Plus HRP Polymer-kit (mouse/rabbit); Zytomed, Berlin, Germany) at room temperature (RT). For visualization of the immunostaining, substrate solution and the chromogen-3, 3′-diaminobenzidine (DAB; Dako, Hamburg, Germany), were applied for 10 min. Finally, the slides were counterstained with Hemalum and dehydrated in an ascending series of alcohol. Placenta (PAF-AH) and colon (β-catenin) tissue sections served as antibody controls. Both positive and negative controls were included in each IHC experiment to confirm antibody function and choose the adequate dilution factor (Additional file Figure S1).

HCC1937 cells were used for immunocytochemistry. A density of 5 × 10^3^ cells/cm^2^ of cells were seeded in eight well microchambers (Millicell EZ SLIDE 8-well glass, Darmstadt, Germany). *PLA2G7* silencing of HCC1937 cells was performed after 60 h of incubation. Untreated HCC1937 cells served as the control group. Subsequently, all slides were washed with PBS 0.1 M, set in a 100% ethanol/methanol (1:1) solution for 15 min at RT, and air-dried. To reduce non-specific antibody binding, the slides were incubated with a protein block (Dako, Glostrup, Denmark) for 20 min at RT. Next, the slides were incubated with anti-PAF-AH (Cayman, Polyclonal Cay-160603, 1:50 dilution, rabbit IgG, MI, USA) and anti-β-catenin antibodies (Diagnostic Biosystems, Polyclonal Roche-D178frzQ, 1:200 dilution, rabbit IgG, California, USA) for 16 h at 4 °C. Afterwards, the slides were washed again with PBS and incubated with a biotinylated secondary anti-Rabbit antibody (Vector Laboratories, Burlingame, CA, USA) at RT. After 30 min, the slides were washed in PBS and incubated with an avidin–biotin–peroxidase complex (Vector Laboratories, Burlingame, CA, USA) for 30 min at RT. The antigen–antibody complex was visualized with chromogen 3-amino-9-ethyl carbazole (Dako, Glostrup, Denmark) and counterstained with Mayer´s hemalum. Finally, all slides were washed in tap water and coverslipped using Kaiser’s glycerin gelatine (Merck, Darmstadt, Germany).

### 4.4. Staining Evaluation

IHC staining results were evaluated by two different researchers in a double-blind process using the semi-quantitative immunoreactive score (Immunoreactive Score (IRS), Remmele’s score) [54]. A Leitz photomicroscope (Wetzlar, Germany) was used to characterize the PAF-AH- and β-catenin-specific staining reactions in the nucleus, the cytoplasm, and on the membrane of the BC cells. Staining intensity and distribution pattern were assessed with the IRS. To obtain the IRS, the optional staining intensity (0: no, 1: weak, 2: moderate, and 3: strong staining) was multiplied with the percentage of stained cells (0: no staining, 1: <10% staining of the cells, 2: 11–50% of the cells, 3: 51–80% of the cells, and 4: >81%). The median IRS from three representative areas of each slide were calculated and used for further analyses.

### 4.5. Statistical Analysis

Statistical analysis was performed by using SPSS 25.0 (v25, IBM, Armonk, NY, USA). The distribution of clinical-pathological variables was evaluated with the Chi-Square test, while the Mann–Whitney-U-test [55] was used to compare IRS between different clinical and pathological subgroups. Spearman analysis [56] was applied to calculate correlations. *p* values less than 0.05 were considered statistically significant. The Ct values of each gene were obtained by qPCR, and the relative gene expression was calculated using the 2^−ΔΔCt^ formula [57]. Figures were generated using Graph Pad Prism 7.03 (v7, La Jolla, CA, USA).

### 4.6. Cell Lines

The human BC cell lines MDAMB231 (triple negative, BRCA1 wildtype), HCC1937 (triple negative, BRCA1 mutant type), MCF7 (Luminal A type), BT474 (Luminal B type), SKBR3 (HER2+), and the breast epithelial cell line MCF10A were purchased from the American Type Culture Collection (ATCC, Rockville, MD, USA). Malignant cells were thawed and maintained in RPMI 1640 medium (ThermoFisher Scientific, Waltham, MA, USA) supplemented with 10% FBS. MCF10A were thawed and maintained in culture with the medium listed in the Additional file (Table S1). All cell lines were grown in a humidified incubator at 37 °C under 5 % CO_2_.

### 4.7. Real-Time PCR

mRNA isolation was performed using the RNeasy Mini Kit (Qiagen, Venlo, The Netherlands) according to the manufacturer’s protocol. After isolation, the reverse transcription was carried out with the MMLV Reverse Transcriptase 1st-Strand cDNA Synthesis Kit (Epicentre, Madison, WI, USA). Therefore, 1 µg RNA was converted into first-strand cDNA according to the manufacturer’s instructions. PCR was performed individually on each sample. The basal and post-transfection mRNA expression levels of PAF-AH were quantified by qPCR using FastStart Essential DNA Probes Master and gene-specific primers (Roche, Basel, Switzerland (Additional file Table S2)). mRNA quantification was achieved according to the 2^−ΔΔCt^ method using β-actin and GAPDH as housekeeping genes. Gene silencing was used to determine PLA2G7 efficiency; the remaining PAF-AH expression was compared to its expression in HCC1937 cells transfected with scrambled siRNA (control).

### 4.8. siRNA Gene Knockdown

Lipofectamine RNAiMAX reagent (Invitrogen, Carlsbad, CA, USA) transfected small interfering RNA-PLA2G7 (QIAGEN Sciences, Maryland, USA) into HCC1937 cells by using two different SiRNA specific for PLA2G7. A scrambled siRNA (Qiagen, Hilden, Germany) served as control. Transfection was carried out according to the manufacturer’s instructions.

Initially, HCC1937 cells were seeded into 6-well plates and finally transfected as soon as their cell density reached 60–70%. After an incubation time of 60 h, the cells were harvested and used for further experiments.

### 4.9. Western Blot

A detailed protocol for Western Blot analysis has previously been published by Tremmel et al. [58]. Briefly, all cells were lysed for 15 min at 4 °C in 200 µL RIPA buffer solution (Sigma-Aldrich Co., St. Louis, MO, USA) containing a protease inhibitor in a 1:100 dilution (Sigma-Aldrich Co., St. Louis, MO, USA). Protein concentrations in cell lysates were quantified via Bradford Assays [59]. Protein extracts (65 µg) were separated according to their molecular weight using a 12% sodium dodecyl sulfate (SDS)-polyacrylamide gel (PAGE) and transferred onto a polyvinylidene fluoride membrane (EMD Millipore, Billerica, MA, USA). The membrane was blocked for 1 h in a 1 × casein solution (Vector Laboratories, Burlingame, CA, USA) to prevent nonspecific binding of the antibodies. After casein saturation, the membrane was stained with primary antibodies overnight at 4 °C. Anti-PAF-AH (proteinTECH, Polyclonal 15526-1-AP, 1:200 dilution, rabbit IgG, Manchester, UK) and anti-GAPDH (GeneTex Co., Monoclonal GT-239, 1:1000 dilution, mouse IgG; Eching, Germany) primary antibodies were used on each membrane. GAPDH Western Blots served as controls. Next, all membranes except the PAF-AH loaded membrane were washed thrice in a 1:10 casein solution and subjected to biotinylated anti-mouse IgG antibodies and ABC-AmP reagent (both VECTASTAIN ABC-AmP Kit for rabbit IgG; Vector Laboratories), whilst the PAF-AH antibody membrane was incubated with biotinylated anti-rabbit IgG antibody and ABC-AmP reagent (both VECTASTAIN ABC-AmP Kit for rabbit IgG; Vector Laboratories). Lastly, the antibody complexes were visualized with a 5-bromo-4-chloro-3-indolylphosphate/nitroblue tetrazolium chromogenic substrate (Vectastain ABC-AmP Kit; Vector Laboratories). Western Blot detection and analysis were performed with Bio-Rad Universal Hood II and Quantity One Software (Bio-Rad Laboratories Inc., Hercules, CA, USA). Each Western Blot experiment was validated nine times (n = 9, three times in three lanes).

### 4.10. Cell Viability Assays

MTT assays were performed to measure cell viability. Therefore, HCC1937 cells were seeded on a 96 well-plate containing with 5000 cells/100 µL. After 48 h of incubation in RPMI 1640 with 10% FBS, transfection was carried out as previously described. Next, cells were incubated in 20 µL MTT (3-(4,5-dimethylthiazol-2-yl)-2,5-diphenyltetrazolium bromide) solution (Sigma-Aldrich Co.) at a concentration of 5 mg/mL for 2 h. The solution was discharged, and the precipitated formazan was dissolved in 200 µL dimethyl sulfoxide (DMSO). Cell viability was measured with an Elx800 universal Microplate Reader at a wavelength of 595 nm. Each experiment was repeated three times (n = 3).

### 4.11. Proliferation Assay

HCC1937 cells were seeded on 96 well-plate and transfected to measure the cell proliferation rate. The 5-Bromo-2’-Deoxyuridine (BrdU) incorporation assay (Roche, Basel, Switzerland) was used according to the manufacturer’s protocol. The optical density was measured at 450 nm using Elx800 universal Microplate Reader. Each experiment was repeated three times (n = 3).

### 4.12. Wound Healing Assay

HCC1937 cells were seeded on a 24 well-plate (2 × 10^5^ cells / ml). After 24 h, the cell monolayer was disrupted by a scratch inflicted by a 100 µL pipet tip. After this, the transfection was carried out, and digital images of the cell layer were taken at 0 h and 60 h after PLA2G7 gene knockdown. Cell migration was monitored using an inverse phase contrast microscope (Leica Dmi1; Leica, Wetzlar, Germany) with a LEICA MC120 HD camera (Leica, Wetzlar, Germany). Microphotographs of wounded areas and areas covered with cells were analyzed by Image J (https://imagej.nih.gov/ij/ accessed on 8 February 2021). The cell migration area is defined as the area difference at the time of transfection and 60 h later.

## 5. Conclusions

In accordance with our previous findings in OC, we observed an association between BRCA1 mutation and PAF-AH expression in BC as well. Functional in vitro experiments showed a protective role of PAF-AH in BRCA1 mutated BC and suggested a negative regulatory impact of PAF-AH on the canonical Wnt signaling pathway. However, the molecular interaction between PAF-AH and β-catenin remains unclear, raising the need for further molecular studies.

## Figures and Tables

**Table 1:** Correlation between PAF-AH and the Wnt signaling protein β-catenin.

	PAF-AH Nucleus	PAF-AH Cytoplasm
**β-catenin nucleus**		
*Cc*	−0.062	0.018
*P*	>0.05	>0.05
*N*	44	44
**β-catenin membrane**		
*Cc*	0.766	0.057
*P*	<0.001 *	>0.05
*N*	44	44

IRScores of PAF-AH (nucleus and cytoplasm) and β-catenin (membrane) staining were correlated using Spearman’s correlation analysis. Significant results are indicated by asterisks (*: *p* ≤ 0.05). Cc = correlation coefficient, P = two-tailed significance, N = number of patients.

**Table 2:** Clinicopathological characteristics of breast cancer patients.

	Clinicopathological Characteristics of Patients		
		n (121)	Percentage [%]
Histology	Invasive ductal	80	66.1
	Invasive lobular	13	10.7
	Invasive medullary	12	9.9
	Invasively mucinous	1	0.8
	Unknown	15	11.8
Grading	1	4	3.3
	2	45	37.1
	3	60	49.6
	Unknown	12	9.9
Age	≥60	29	24.0
	<60	92	76.0
Primary tumor expansion	Tis	5	4.1
	T1a	3	2.5
	T1b	13	10.7
	T1c	22	21.5
	T2	43	36.2
	T3	12	9.9
	T4a	2	1.6
	T4b	2	1.6
	T4c	1	0.8
	T4d	6	5.0
	Tx	12	9.9
Nodal status	N0	38	31.4
	N1	34	28.1
	N2	9	7.4
	N3	7	5.8
	Nx	33	27.3
Distant Metastasis	M0	46	38.0
	M1	28	23.1
	Mx	47	53.5
BRCA1 mutation status	No BRCA1 mutation	28	23.1
	BRCA1 mutation	22	18.2
	Unknown	71	58.7

## Data Availability

The datasets generated and/or analyzed during the current study are available from the corresponding author on reasonable request.

## Supplementary Materials

The following are available online at https://www.mdpi.com/1422-0067/24/1/882/s1, Table S1: Cell culture components for MCF10A, Table S2: Sequences of primers used in qPCR to determine mRNA expression levels, Figure S1: Positive and negative controls for each antibody.
