# Supplementary material for: PLA2G7/PAF-AH as Potential Negative Regulator of the Wnt Signaling Pathway Mediates Protective Effects in BRCA1 Mutant Breast Cancer
Source: Int J Mol Sci. 2023 Jan 3;24(1):882. doi: 10.3390/ijms24010882 (PMC9821466; doi:10.3390/ijms24010882)
Supplement: Supplementary file 1 [file ijms-24-00882-s001.zip › ijms-2002918-supplementary.pdf]

Additional file to

PLA2G7/PAF-AH as potential negative regulator of the Wnt signaling pathway mediates protective effects in BRCA1 mutant breast cancer

Table S1: Cell culture components for MCF10A.

Component	Growth Medium	Assay Medium (Without EGF)
DMEM/F12 (Invitrogen #11330-032)	500.0 ml	500.0 ml
Horse Serum (Invitrogen#16050-122)	25.0 ml (5% final)	10.00 ml (2% final)
EGF (100 µg/ml stock) (Peprotech, 1 mg)	100 µl (20ng/ml final)	--
Hydrocortisone (1mg/ml) (Sigma #H-0888)	250 µl (0.5 mg/ml final)	250 µl (0.5 µg/ml final)
Cholera Toxin (1mg/ml stock) (Sigma #C-8052)	50 µl (100 ng/ml final)	50µl (100 ng/ml final)
Insulin (10mg/ml stock) (Sigma #I-1882)	500 µl (10µg/ml final)	500 µl (10µg/ml final)
Pen/Strep (100 x solution, Invitrogen #15070-063)	5.0 ml	5.0 ml

Table S2: Sequences of primers used in qPCR to determine mRNA expression levels.

PLA2G7	Forward: GGCTCTACCTTAGAACCCTGAAA<br>Reverse: TTTTGCTCTTTGCCGTACCT
ACTB	Forward: TCCTCCCTGGAGAAGAGCTA<br>Reverse: CGTGGATGCCACAGGACT
GAPDH	Forward: AGCCACATCGCTCAGACAC<br>Reverse: GCCCAATACGACCAAATCC

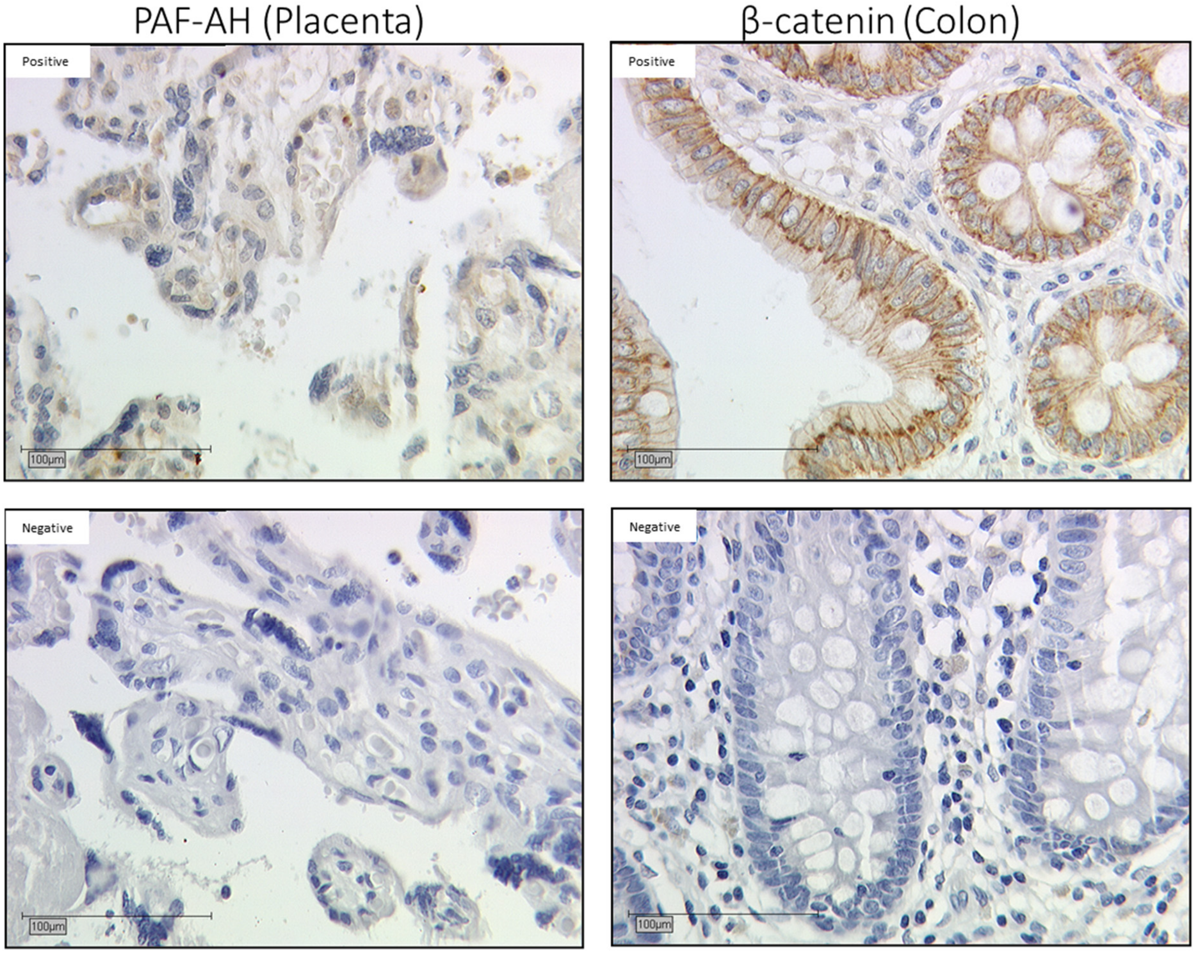

Figure S1: Positive and negative system controls for PAF-AH and β-catenin staining. Placenta and colon tissue served as positive and negative system controls for immunostainings (scale bar = 100µm).
